# Cardiac Memory-induced T-wave Inversions

**DOI:** 10.5811/cpcem.2020.1.45527

**Published:** 2020-04-14

**Authors:** Sara C. Polito, Jonathan A. Giordano, Benjamin L. Cooper

**Affiliations:** McGovern Medical School at the University of Texas Health Science Center at Houston (UTHealth), Department of Emergency Medicine, Houston, Texas

**Keywords:** ECG, cardiac memory, T-wave inversions

## Abstract

**Introduction:**

Cardiac memory refers to T-wave inversions that result when normal ventricular activation resumes following a period of abnormal ventricular activation.

**Case Report:**

We present a case of a 29-year-old man with a pacemaker who presented with new, deep symmetric T-wave inversions caused by cardiac memory.

**Discussion:**

Abnormal ventricular activation is most commonly induced by ventricular pacing but can also occur in the setting of transient left bundle branch blocks, ventricular tachycardia, and intermittent ventricular pre-excitation.

**Conclusion:**

Recognition of this phenomenon may help to reduce unnecessary admissions, cardiac testing, and cardiac catheterizations.

## INTRODUCTION

The differential for new T-wave inversions (TWI) includes myocardial ischemia, ventricular overload syndromes (i.e., strain), Takotsubo cardiomyopathy, myopericarditis, and cerebrovascular injury.^1^ Here we present a case of deep, symmetric TWI induced by a phenomenon known as cardiac memory. Cardiac memory is characterized by transient T-wave inversion after a period of abnormal ventricular activation. It is generally considered to be a benign finding, in contrast to many other causes of TWIs, although other etiologies should be ruled out prior to diagnosing cardiac memory.

## CASE REPORT

**Image 1:**
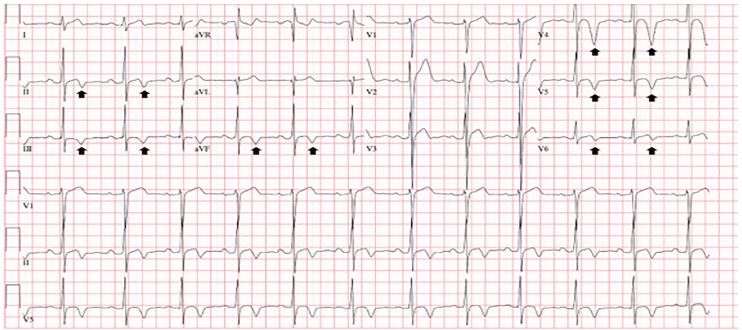
Initial electrocardiogram on presentation revealing deep and symmetric T-wave inversions in the inferior leads (II, III, aVF) and precordial leads V4–V6 (arrows).

A 29-year-old man with a history of second-degree Mobitz type II atrioventricular block and a dual-chamber right ventricular pacemaker (placed five weeks prior to presentation) presented to the emergency department for one week of sharp, intermittent, unprovoked, left-sided chest pain over his pacemaker site. On arrival, his pulse rate was 78 beats per minute, blood pressure 171/99 millimeters of mercury, respiratory rate 16 breaths per minute, oxygen saturation 98% on room air, and temperature 98.3º Fahrenheit (36.7º Celsius). The patient appeared comfortable and had reproducible chest pain over the pacemaker site. A 12-lead electrocardiogram (ECG) was obtained (Image 1).

**Image 2:**
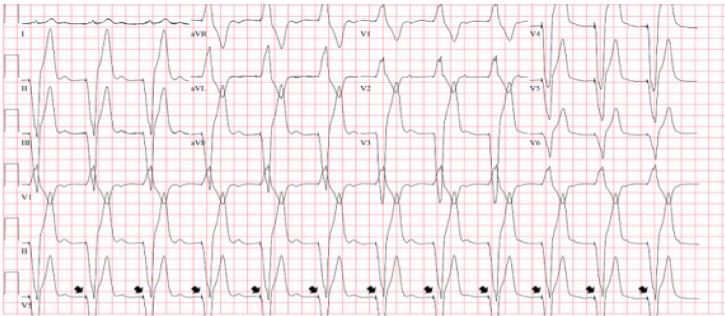
The electrocardiogram obtained four hours after presentation revealed a paced rhythm (pacing spikes demonstrated by arrows).

Electrolytes and complete blood count were within normal limits, and the troponin I level was undetectable. The patient was given 324 milligrams of aspirin, started on a heparin drip, and admitted to the coronary care unit for further work-up. A repeat ECG performed four hours after presentation showed a ventricular-paced rhythm (Image 2).

**Image 3:**
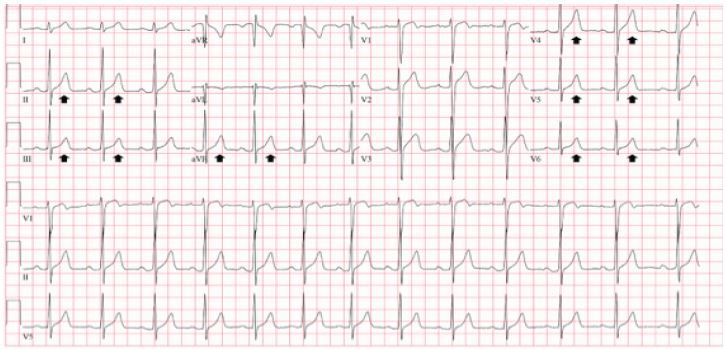
Baseline electrocardiogram obtained one month prior to presentation, similar to the electrocardiogram performed two months later, showing resolution of T-wave inversions (arrows).

The troponin I level remained undetectable for three serial measurements spanning 11 hours, and the heparin drip was discontinued. A transthoracic echocardiogram showed normal left ventricular size and function without wall motion abnormalities, and mild concentric hypertrophy. The pacemaker was interrogated, revealing adequate function and battery life. An ultrasound of the left chest wall excluded a hematoma or fluid collection around the pacemaker. He was started on oral nifedipine for newly diagnosed hypertension, remained asymptomatic and was discharged the following morning. A repeat ECG two months later revealed a return to his normal baseline T-wave morphology (Image 3).

## DISCUSSION

While the presenting history and physical examination were not alarming, the initial ECG finding of deep and symmetric TWIs prompted concern for acute coronary syndrome. The differential diagnosis of new TWIs includes myocardial ischemia, ventricular overload syndromes (i.e., strain), Takotsubo cardiomyopathy, myopericarditis, and cerebrovascular injury.^1^ Morphologic features of TWIs that prompt concern for acute coronary syndrome include narrow, deep amplitude and symmetry.^1^,^2^ The TWIs in the presenting ECG would certainly meet these criteria. The TWIs were new when compared to a prior ECG (Image 3); however, serial undetectable troponin I levels, an echocardiogram failing to reveal wall motion abnormalities, lack of concerning historical elements, and reproducible chest wall pain at the pacemaker site all refuted coronary ischemia. The distribution of the TWIs in the setting of recent ventricular pacing was most consistent with cardiac memory.

**CPC-EM Capsule**

What do we already know about this clinical entity?

Cardiac memory refers to T-wave inversions (TWI) that result when normal ventricular activation resumes following a period of abnormal ventricular activation.

What makes this presentation of disease reportable?

Deep and symmetric TWIs prompted concern for acute coronary syndrome, but the distribution of the TWIs in the setting of recent ventricular pacing is most consistent with cardiac memory.

What is the major learning point?

Cardiac memory refers to T-wave changes that result when normal ventricular activation resumes and is most commonly induced by ventricular pacing.

How might this improve emergency medicine practice?

Recognition of this phenomenon may help to reduce unnecessary admissions, cardiac testing, and cardiac catheterizations.

Cardiac memory refers to T-wave changes that result when normal ventricular activation resumes following a period of abnormal ventricular activation (i.e., wide QRS complexes). Abnormal ventricular activation is most commonly induced by ventricular pacing, but can also occur in the setting of transient left bundle branch blocks (LBBB), ventricular tachycardia, and intermittent ventricular pre-excitation.^3^,^4^ The T-wave “remembers” the vector of prior abnormal ventricular activation and reflects the direction of the wide QRS complexes after restoration of normal ventricular activation.^4^,^5^ This results in positive T-waves in leads that had wide, positive QRS complexes during abnormal activation, and negative T-waves in leads that had wide, negative QRS complexes.

It has been shown that the duration of the T-wave changes persists for longer periods of time with increasing duration of the abnormal ventricular activation. For example, when human subjects were electrically paced for 10 minutes, transient TWIs lasted for 15 minutes after pacing was stopped. However, when paced for two years, TWIs persisted for up to 18 months. The prevailing mechanism for the development of cardiac memory is thought to involve mechanical myocyte stretch leading to increased angiotensin II and subsequent internalization of a subunit of transient outward potassium channels (I_to_). I_to_ channels are responsible for the outward flow of potassium following depolarization; therefore, disruption of this process leads to repolarization abnormalities and morphologic changes of the T-wave.^6^

Identification of TWIs caused by cardiac memory may help to reduce unnecessary admissions, cardiac testing, and cardiac catheterizations.^4^,^6^ Certain characteristics in the T-wave can help to differentiate TWI caused by cardiac memory from those caused by ischemia. Shvilkin et al found 92% sensitivity and 100% specificity for diagnosing cardiac memory when the following two criteria are met:

1. A positive T-wave in aVL combined with a positive or isoelectric T-wave in lead I
2. Precordial TWI with larger magnitude than any TWI in lead III.^3^

Additionally, having access to an ECG with a preceding wide QRS can help diagnose cardiac memory by comparing the location of the wide QRS complexes during abnormal activation to those of the TWI during sinus rhythm.^4^ However, the diagnosis of cardiac memory should only be made after other causes of TWI are reliably ruled out.^4^,^6^

Although cardiac memory has traditionally been thought of as benign, recent studies have called this into question.^5^,^6^ There are multiple case reports of torsades de pointes (TdP) in patients who experience cardiac memory. Changing from a pattern of abnormal ventricular activation to sinus rhythm can prolong the QT interval, placing patients at risk for TdP.^5^,^6^ Additionally, in patients without pacemakers, TWIs caused by cardiac memory may indicate intermittent LBBB, paroxysmal ventricular tachycardia, or intermittent ventricular preexcitation, which may be important considerations, especially in patients presenting with syncope, presyncope, or palpitations.^6^

## CONCLUSION

Cardiac memory is a pattern of TWIs that occurs following resolution of wide QRS complexes. While most commonly seen in patients with ventricular pacemakers, it can be seen with intermittent LBBB, ventricular tachycardia, and intermittent ventricular preexcitation. Cardiac memory should not be diagnosed as the cause of TWI until other causes are reliably ruled out, but recognition of this phenomenon may help to reduce unnecessary admissions, cardiac testing, and cardiac catheterizations.
